# Supplementary material for: Morphometric convergence between Proterozoic and post-vegetation rivers
Source: Nat Commun. 2017 May 26;8:15250. doi: 10.1038/ncomms15250 (PMC5458558; doi:10.1038/ncomms15250)
Supplement: Supplementary Information — Supplementary Figures [file ncomms15250-s1.pdf]

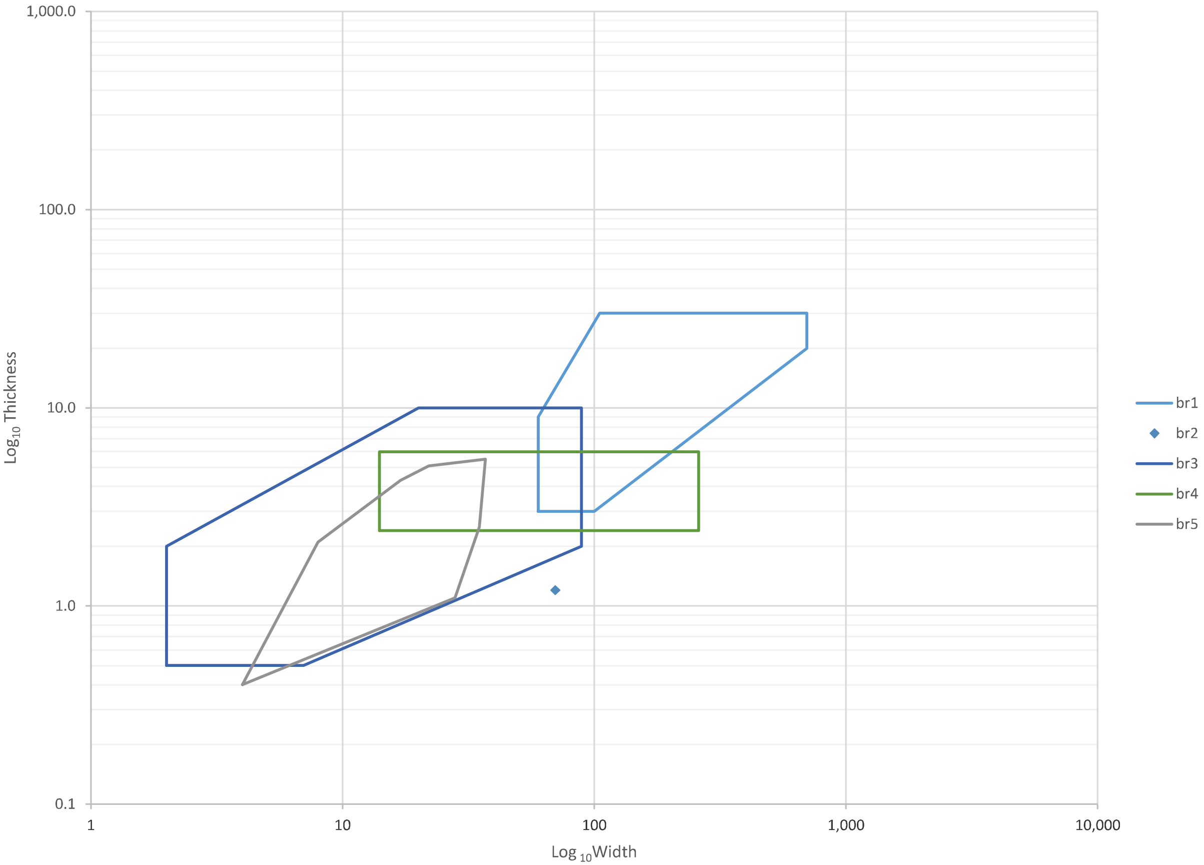

Supplementary Figure 1 | Geometry of low-sinuosity channel forms (Ordovician-Pleistocene)

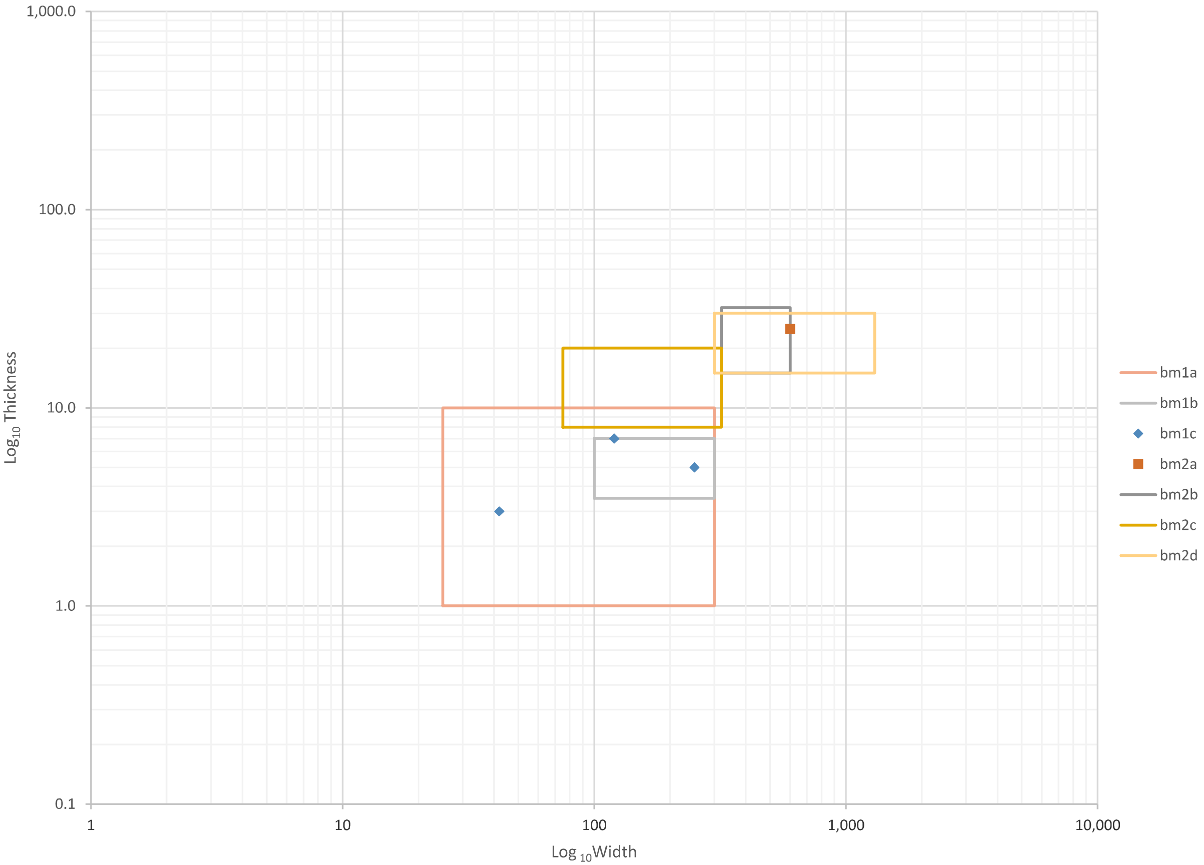

Supplementary Figure 2 | Geometry of intermediate-sinuosity channel forms (Ordovician-Pleistocene)

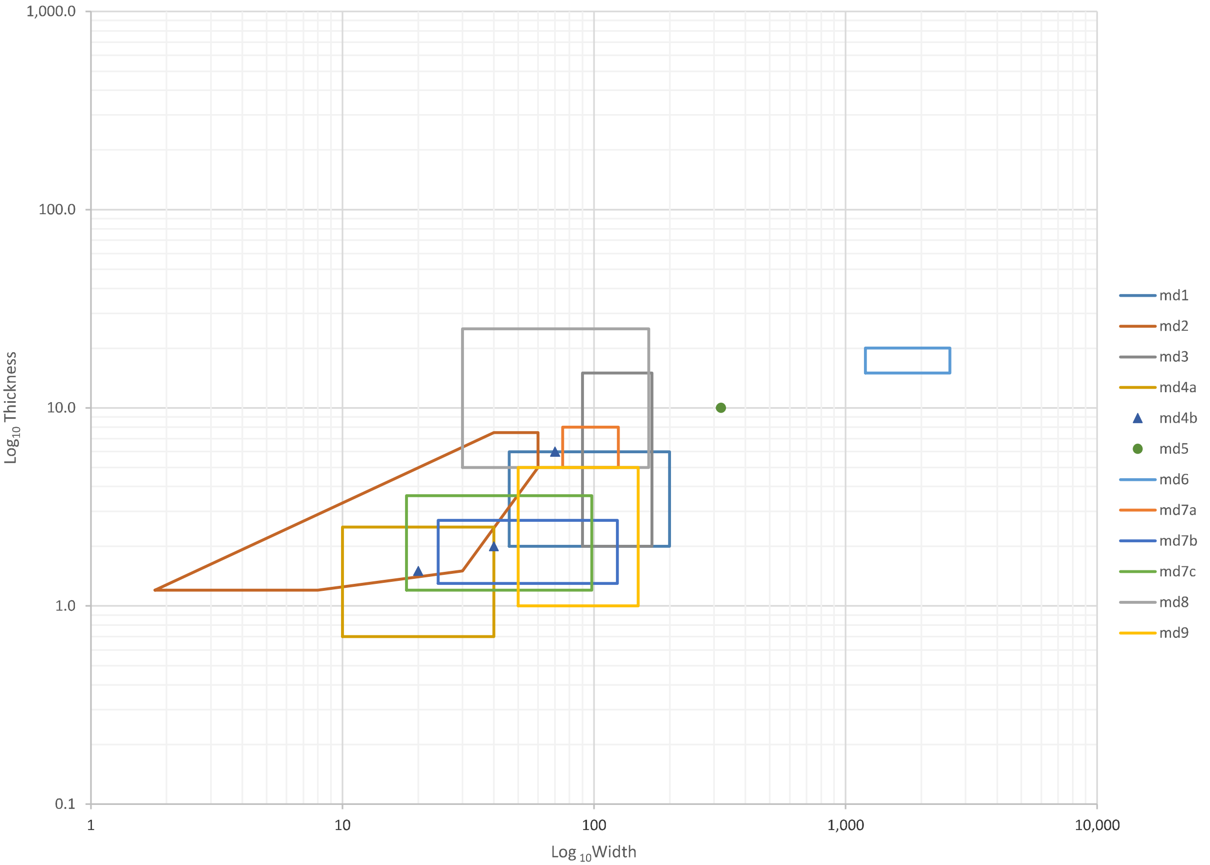

Supplementary Figure 3 | Geometry of high-sinuosity channel forms (Ordovician-Pleistocene)

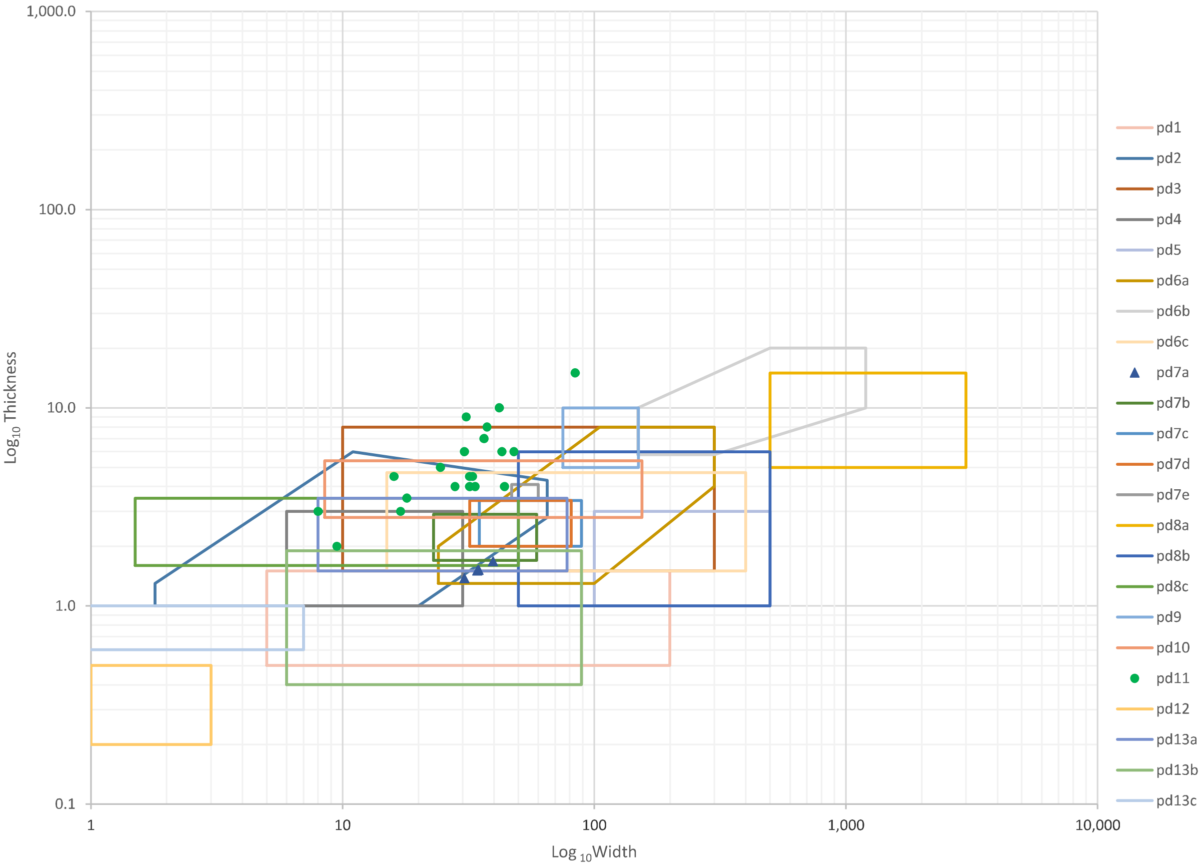

Supplementary Figure 4 | Geometry of distributary channel forms (Ordovician-Pleistocene)

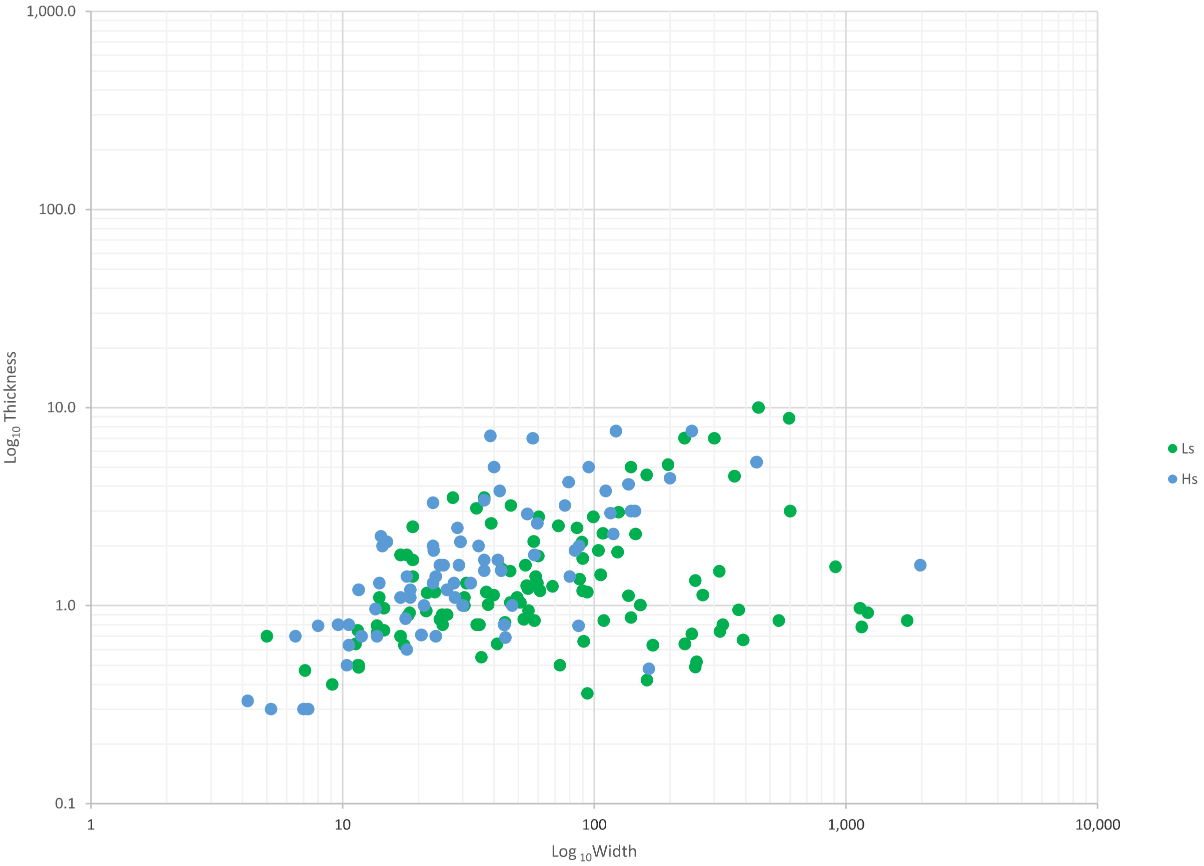

Supplementary Figure 5 | Geometry of modern, perennial river cross-sections (Ordovician-Pleistocene)
